# Review and update of temporal bone imaging

**DOI:** 10.1590/0100-3984.2019.52.2e2

**Published:** 2019

**Authors:** Regina Lúcia Elia Gomes

**Affiliations:** 1 Physician at the Department of Radiology of the Instituto de Radiologia do Hospital das Clínicas da Faculdade de Medicina da Universidade de São Paulo (In-Rad/HC-FMUSP), and at the Hospital Israelita Albert Einstein, São Paulo, SP, Brazil. E-mail: regina.gomes@hc.fm.usp.br.

The evaluation of the temporal bones by imaging methods is challenging and thought-provoking, requiring that the radiologist have specific knowledge of the anatomy of the many small, delicate local structures. One interesting aspect is that the imaging findings can be symmetric and bilateral, making recognition of the normal anatomy even more important. The correlation with clinical data is also essential.

An article published in the previous issue of Radiologia Brasileira^(^^1^^)^ presents a review of the imaging findings of the main hearing disorders, all of which have a great impact on patient quality of life, together with a brief summary of the clinical presentation of each. The article in question, authored by Salata et al.^(^^1^^)^, divides hearing disorders by etiology-neoplastic, infectious/inflammatory, congenital, traumatic/post-surgical, or other-which is the ideal didactic approach for a pictorial essay. Another way of analyzing the temporal bone is by anatomical division. It is important for radiologists to create a logical sequence in their routine evaluation, so that they do not overlook any of the structures. The typical approach is to start from the outside and work inward, evaluating the external ear, external auditory canal, mastoid, middle ear, inner ear, petrous apex, facial nerves, vestibulocochlear nerves, and extra-axial regions of the brain, such an approach being crucial for formulating a diagnostic hypothesis and making the differential diagnosis^(^^2^^)^.

Among the imaging methods currently available, the most widely used are computed tomography (CT) and magnetic resonance imaging (MRI), both of which are represented in this article, as the means of illustrating the various diseases mentioned. It is important that radiologists know the main indications for the use of each modality, as well as its limitations^(^^3^^)^.

In brief, CT allows excellent detection of calcifications, cortical bone, air, and fat, particularly in the study of the external and middle ears, as well as the otic capsule^(^^3^^)^. The recently developed modality known as cone-beam CT provides higher resolution in temporal bone examinations and exposes patients to less radiation, although its indications are quite limited, as is its availability in Brazil. However, it has been used for monitoring the evolution of alterations already identified on multidetector CT and for the diagnosis of pathologies in children, as well as for the evaluation of ossicular prostheses, cochlear implants, malformations, and otosclerosis^(^^4^^,^^5^^)^.

MRI offers excellent characterization of the soft tissues, being indicated for the investigation of masses in the middle ear and of alterations that affect nerve structures such as those in the inner ear, middle cerebral fossa, posterior cerebral fossa, and base of the skull^(^^3^^)^. In addition, new MRI techniques, such as diffusion-weighted and perfusion-weighted imaging, are already part of the routine diagnostic protocol at various centers, especially diffusion-weighted imaging for the preoperative (surgical planning) or postoperative (follow-up) evaluation of cholesteatoma, which classically presents restricted diffusion^(^^6^^)^, although lesions smaller than 3 mm might not be detected, even in 3.0 T scanners.

Another recently developed MRI technique, currently used at only a few centers around the world^(^^4^^,^^6^^-^^8^^)^, has cast new light on the diagnosis of Ménière's disease (MD). It consists in performing MRI in a 3.0 T scanner 4 h after intravenous administration of paramagnetic contrast medium, employing a three-dimensional fluid-attenuated inversion recovery sequence for the diagnosis and grading of endolymphatic hydrops, which is present in up to 73% of patients with clinically possible MD, in 100% of those with clinically probable MD, and in 95% of those with clinically definite MD. Although not yet a diagnostic criterion, as a future perspective and with optimization of the technique, it could contribute to the diagnosis, influence therapeutic decision making, and play a role in follow-up evaluations^(^^6^^-^^8^^)^.

In conclusion, the Salata et al.^(^^1^^)^ article presents a comprehensive review of the hearing disorders that have a great impact on patient quality of life. It is fundamental that radiologists have knowledge of the anatomical structures of the temporal bones and create a routine for reporting their analysis of an examination, as well as being aware of the newest techniques available, in order to select the imaging method best suited to each clinical situation.
